# Taxonomic status of *Populuswulianensis* and *P.ningshanica* (Salicaceae)

**DOI:** 10.3897/phytokeys.108.25600

**Published:** 2018-09-10

**Authors:** Lei Zhang, Mingcheng Wang, Tao Ma, Jianquan Liu

**Affiliations:** 1 Key Laboratory of Bio-Resource and Eco-Environment of Ministry of Education, College of Life Sciences, Sichuan University, Chengdu 610065, Sichuan, P. R. China Sichuan University Chengdu China

**Keywords:** Geometric morphometrics, microsatellites, DNA barcodes, integrated species delimitation

## Abstract

Species delimitation in the genus *Populus* is particularly challenging due to high levels of intraspecific polymorphism as well as frequent interspecific hybridisation and introgression. In this study, we aimed to examine the taxonomic status of *Populusningshanica* and *P.wulianensis* using an integrative taxonomy that considers multiple operational criteria. We carried out morphometric analyses of leaf traits and genetic examinations (including sequence variations at five barcoding DNAs and polymorphisms at 14 nuclear microsatellite SSR primers) at the population level between them and two closely related species *P.adenopoda* and *P.davidiana*. Results suggest that *P.wulianensis* belongs to the polymorphic species, *P.adenopoda* and should be considered as a synonym of the latter. *P.ningshanica* may have arisen as a result on the hybridisation between *P.adenopoda* and *P.davidiana* and therefore should be treated as *P.×ningshanica*. This study highlights the importance of the integrated evidence in taxonomic decisions of the disputed species.

## Introduction

Species delimitation is essential to conserve and assess biodiversity (Agapow et al. 2004). Any incorrect species recognition may result in serious after-effects in related studies, for example, by an increase in species conservation (Wiens 2007) and under- or over-estimation of biodiversity (Douady 2007). Therefore, in addition to morphological traits, significant efforts have been made to delimit species based on DNA sequence variation (Wiens and Penkrot 2002; Sites and Marshall 2003; Kress et al. 2005; Bond and Stockman 2008; Fujita et al. 2012; Hendrixson et al. 2013) or other genetic polymorphisms that can assess gene flow and identify interspecific hybrids according to the biological species concept (Pérez-Losada et al. 2005). These molecular markers have been used to differentiate species, hybrids and even clones in the genus *Populus* (Salicaceae) (Hamzeh and Dayanandan 2004; Cervera et al. 2005; Hamzeh et al. 2006; Fladung and Buschbom 2009; Schroeder et al. 2012; Feng et al. 2013; Wan et al. 2013). Poplars are widely distributed in the Northern Hemisphere with an important ecological role in natural and artificial forests in both boreal and temperate regions (Dickmann et al. 2001). However, due to high levels of morphological variation and extensive inter-specific hybridisation, species delimitation within the genus is highly contentious (Eckenwalder 1996; Dickmann and Kuzovkina 2008). The number of the proposed species ranges from 22 to 85, plus hundreds of hybrids, varieties and cultivars (Dickmann and Stuart 1983; Fang et al. 1999). Numerous described species were doubted as being hybrids of the other independently evolving lineages (good species) or intra-specific variations of the polymorphic species. However, these ambiguous species have not been well examined.

In this study, we aimed to determine the taxonomic status of two species described from China: *P.wulianensis* S.B.Liang & X.W.Li and *P.ningshanica* C. Wang & Tung (Fang et al. 1999) based on morphometric analyses and genetic examinations at the population level as recently suggested for an integrated species delimitation (Liu 2016). *P.wulianensis* is restricted to eastern Shandong while *P.ningshanica* is distributed in southern Shaanxi and Northwest Hubei. Both are morphologically similar to *P.davidiana* Dode and *P.adenopoda* Maxim. of sect. *Populus* with widespread distributions in northern or middle to southern China. The key traits for their diagnosis are mainly based on leaf characters: blade and apex shape and margin incision (Fang et al. 1999). We firstly conducted morphometric analyses of leaf traits for representative populations of all four species. Then we examined genetic delimitations between them based on evidence from sequence variation of internal transcribed spacer (ITS) and four chloroplast DNA (cpDNA) and genetic polymorphisms from nuclear microsatellite loci (nSSR).

## Materials and methods

### Sample collection

**Figure 1.**
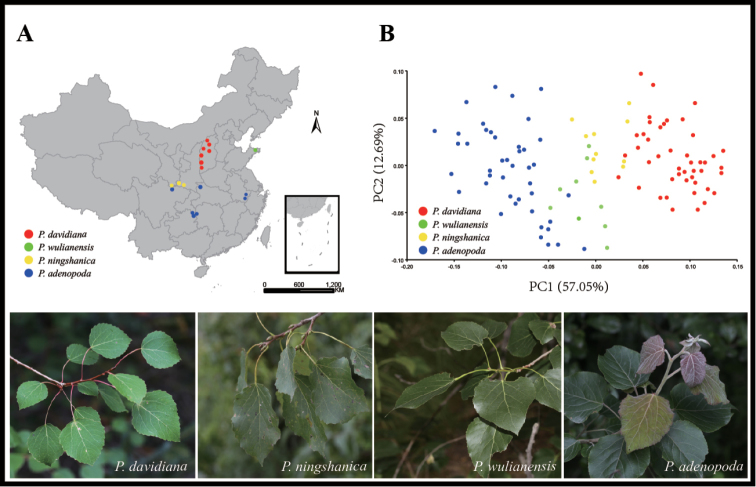
**A** Geographical distribution of 17 populations of the four species (*Populusadenopoda*, *Populusdavidiana*, *Populusningshanica*, *Populuswulianensis*) **B** The Principal Component Analysis (PCA) plot for the morphological variations of 17 populations of 4 species.

We sampled 163 individuals from 17 populations of four species (Table 1), including all recorded natural populations of both *P.ningshanica* and *P.wulianensis*. All individual trees were chosen with typical morphological leaf traits (Fang et al. 1999). Each tree was set apart by at least 50m in each population. Except for collecting specimens (SZ, herbarium of Sichuan University, Chengdu, China) for geometric morphometric analyses, we further selected healthy and fresh leaves from each tree and dried them immediately in silica gel for DNA extraction. We also used an Etrex GIS monitor (Garmin, Taiwan) to record latitude, longitude and altitude of each sampled population (Table 1; Fig. 1A).

**Table 1.** Detailed information for the 17 sampled populations of the sect. *Populus* species that were adopted for Data analysis using SSR and Geometric morphology.

Species	Pop	Individuals	Lon (N)	Lat (E)	Alt (m)	CS	Vouchers
* P. davidiana *	1	21	111.2848	38.21627	1467	Lvliang, SX	LiuJQ-MZL-2013-117
	2	8	111.3395	38.14662	1587	Lvliang, SX	LiuJQ-MZL-2013-121
	3	6	112.3880	38.92512	1402	Qizhou, SX	LiuJQ-MZL-2013-109
	4	10	112.0744	38.8556	1855	Qizhou, SX	LiuJQ-MZL-2013-115
	5	8	111.4328	37.8976	1961	Lvliang, SX	LiuJQ-MZL-2013-124
	6	9	111.2637	37.203483	1459	Lvliang, SX	LiuJQ-MZL-2013-136
* P. ningshanica *	7	8	105.249	32.74979	657	Longnan, GS	LiuJQ-SHX-2015-20
	8	1	107.1394	32.60744	865	Hanzhong, SaX	LiuJQ-SHX-2015-14
	9	3	106.0741	33.55506	768	Hanzhong, SaX	LiuJQ-SHX-2015-10
* P. wulianensis *	10	10	121.7556	37.2983	188	Yantai, SD	LiuJQ-ZL-2016-300
* P. adenopoda *	11	5	108.8565	28.1423	798	Tongren, GZ	MaoKS-CX-2014-326
	12	5	109.1866	28.2958	643	Tongren, GZ	MaoKS-CX-2014-327
	13	5	108.7551	28.3148	707	Tongren, GZ	MaoKS-CX-2014-328
	14	18	105.3035	32.5254	598	Guangyuan, SC	LiuJQ-ZF-2016-01
	15	10	117.8054	30.4742	677	Liuan, AH	LiuJQ-ZF-2016-02
	16	17	117.9531	30.5850	26	Chizhou, AH	LiuJQ-ZF-2016-03
	17	19	110.3215	32.6738	683	Shiyan, HB	LiuJQ-ZF-2016-04

Abbreviations: Pop, Population; Lon (N), Longitude; Lat (E), Latitude; Alt (m), Altitude; CS, Collection site. SX, Shanxi; GS, Gansu; SaX, Shaanxi; SD, Shandong; GZ, Guizhou; SC, Sichuan; AH, Anhui; HB, Hubei.

### Geometric morphometrics

Although we failed to find type specimens of *P.ningshanica* and *P.wulianensis*, we included the newly collected specimens from their type localities. A Canon 60D digital camera was used to photograph typical leaves of all specimens. We transformed every image into a vector diagram using TpsUtil version 1.64 (Rohlf 2013). Thirty-two homologous landmarks were assigned in order to quantify leaf blades shape in all specimens. Landmark positions of leaves included base, tip and margin. All landmarks were digitised for each individual using the software TpsDig version 2.22 (Rohlf 2015). We created a combined data file including all specimens. We implemented morphometrics analyses in MorphoJ version 1.01b (Klingenberg 2011), within which a principal component analysis of morphological variations was conducted and plotted.

### Genetic analyses

We isolated the total genomic DNA from leaves of each individual, based on the hexadecyltrimethyl ammonium bromide (CTAB) method (Doyle 1987). We used a total of 14 SSRs primers (Suppl. material 1: Table S1) developed previously, based on the genome sequences of *Populuseuphratica* and *P.trichocarpa* (Ma et al. 2013; Jiang et al. 2016) to genotype our samples. The PCRs were performed in a volume of 25 ml, which contained: 50–100 ng diluted genomic DNA, 0.5 mM of each dNTP, 0.5 µl of each primer, 2.5 µl 10 × Taq buffer and 0.5 units of Taq polymerase (Vazyme Biotech, Nanjing, China). The PCR programme used was: initially a single cycle at 95 °C for 5 min, followed by 36 cycles at 95 °C for 45s, 55 °C for 40 s and 72 °C for 80 s, with a ﬁnal extension at 72 °C for 10 min. The PCR products at each locus were analysed on an ABI 3830xl DNA analyser (Applied Biosystems, Inc., Foster City CA) at Tsingke Biological Technology (Beijing, China). We used STRUCTURE version 2.3.4 (Falush et al. 2003) that allows a Bayesian hybrid mixture computation to identify genetic compositions of all sampled trees. We pre-assigned a number of genetic clusters (K) ranging from 1 to 10. All runs involved 1,000,000 Markov chain Monte Carlo repetitions after a burn-in period of 500,000 iterations. We used the long burn-in and run lengths as well as 10 replicates to ensure the reproducibility of STRUCTURE results (Gilbert et al. 2012). We estimated the posterior probability of K and Delta K (ΔK), the rate of change of Ln P (K) between successive K values (Evanno et al. 2005). We determined the most likely number of clusters.

We also sequenced internal transcribed spacer (ITS) and four chloroplast DNA (cpDNA) fragments: *matK*, *trnH-psbA*, *trnG-psbK* and *psbK-psbI* for three to five individuals from each sampled population of four species used for nSSR genotyping. In addition, one individual of *P.euphratica* was sequenced as the outgroup. Primers, PCRs and sequencing followed Feng et al. (2013) (Suppl. material S1: Table S2). Sequences for each fragment were aligned and sequences from four cpDNAs were connected using MEGA 7.0 (Kumar et al. 2016). We constructed unrooted neighbour-joining (NJ) trees for both ITS and cpDNAs datasets by MEGA 7.0 (Kumar et al. 2016) respectively, using pairwise deletion and the *P*-distance model. Bootstrap values were estimated with 1000 random addition sequence replicates.

## Results

### PCA analyses of geometric morphometric data

Geometric morphometric analyses of leaf traits yielded 30 principal components (PC), which accounted for all leaf variations. PC1 to PC3 were the only PCs that individually represented >5% of the variance (PC1=57.05%; PC2=12.69%; PC3=7.68%) and they together represented 77.43% of the variance. All other PCs accounted for <5% of the variance individually. The greatest amount of shape variance is observed across PC1 and PC2 (Fig. 1B). Across these two axes, individuals of *P.davidiana* and *P.adenopoda* were treated as a clear division, whereas individuals of *P.wulianensis* and *P.ningshanica* are clustered into one subgroup of the *P.adenopoda* group. All other PCs showed similar relationships.

### Clustering analyses based on the SSR polymorphisms

**Figure 2.**
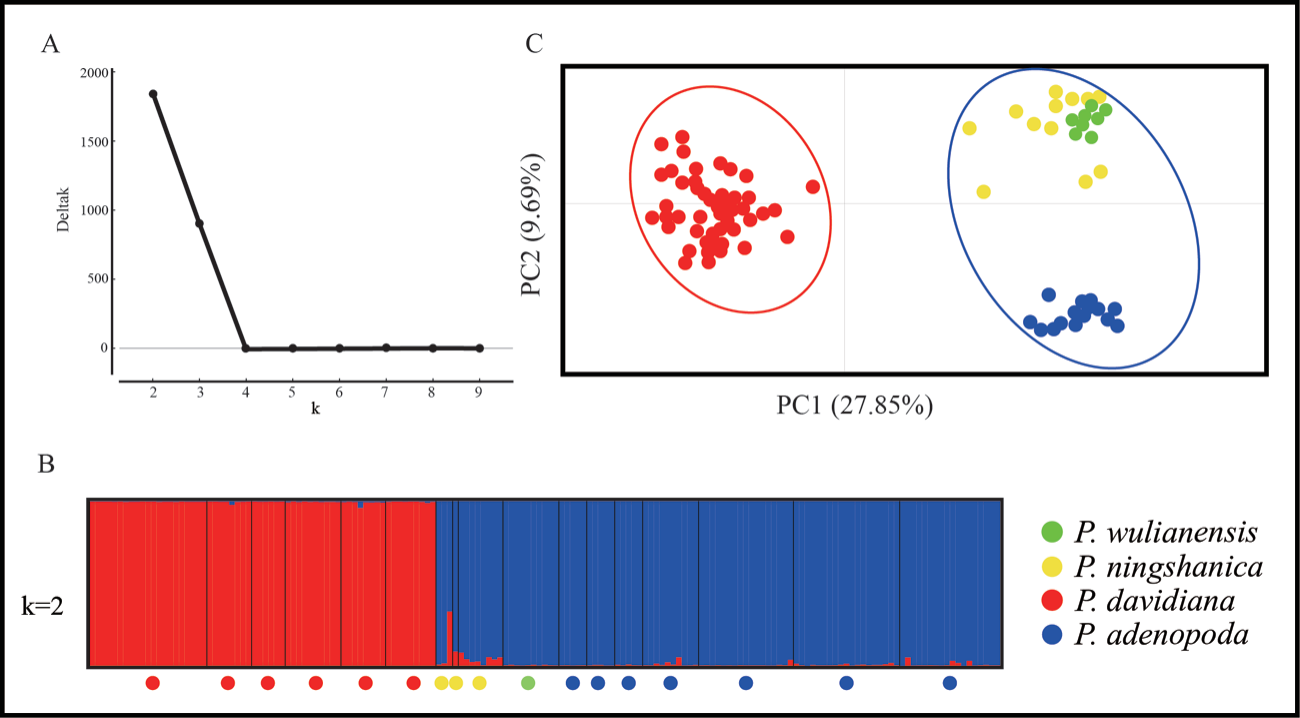
Principal Coordinates Analysis (PCA) of the 17 populations of 4 species based on genetic distance using SSR data (**A**); the optimal K value was estimated using (**B**) the distribution of delta K (*K=2*) and Bayesian clustering plots for 17 populations of 4 species based on variation at 14 nSSR loci (**C**).

We genotyped 14 nuclear SSR loci for 163 sampled individuals of four species. Using the method originally described by Pritchard et al. (Pritchard et al. 2000) and also the ΔK approach described by Evanno et al. (Evanno et al. 2005), we found the most likely number of Bayesian clusters was two (*K* = 2) (Fig. 2A). When *K* = 2, individuals from *P.davidiana* clustered into one group and those from *P.adenopoda* into the other. Within each group, some samples indicated the weak genetic introgression from the other. All sampled individuals of both *P.wulianensis* and *P.ningshanica* were assigned to the group represented by *P.adenopoda* (Fig. 2B). However, approximately 10% of the genetic composition of *P.ningshanica* derived from the cluster represented by *P.davidiana*, while more than 90% was from *P.adenopoda*. Similar results were obtained based on PCA analyses of genetic polymorphisms and that two groups were identified to be, respectively, represented by *P.davidiana* and the other three (Fig. 2C).

**Figure 3.**
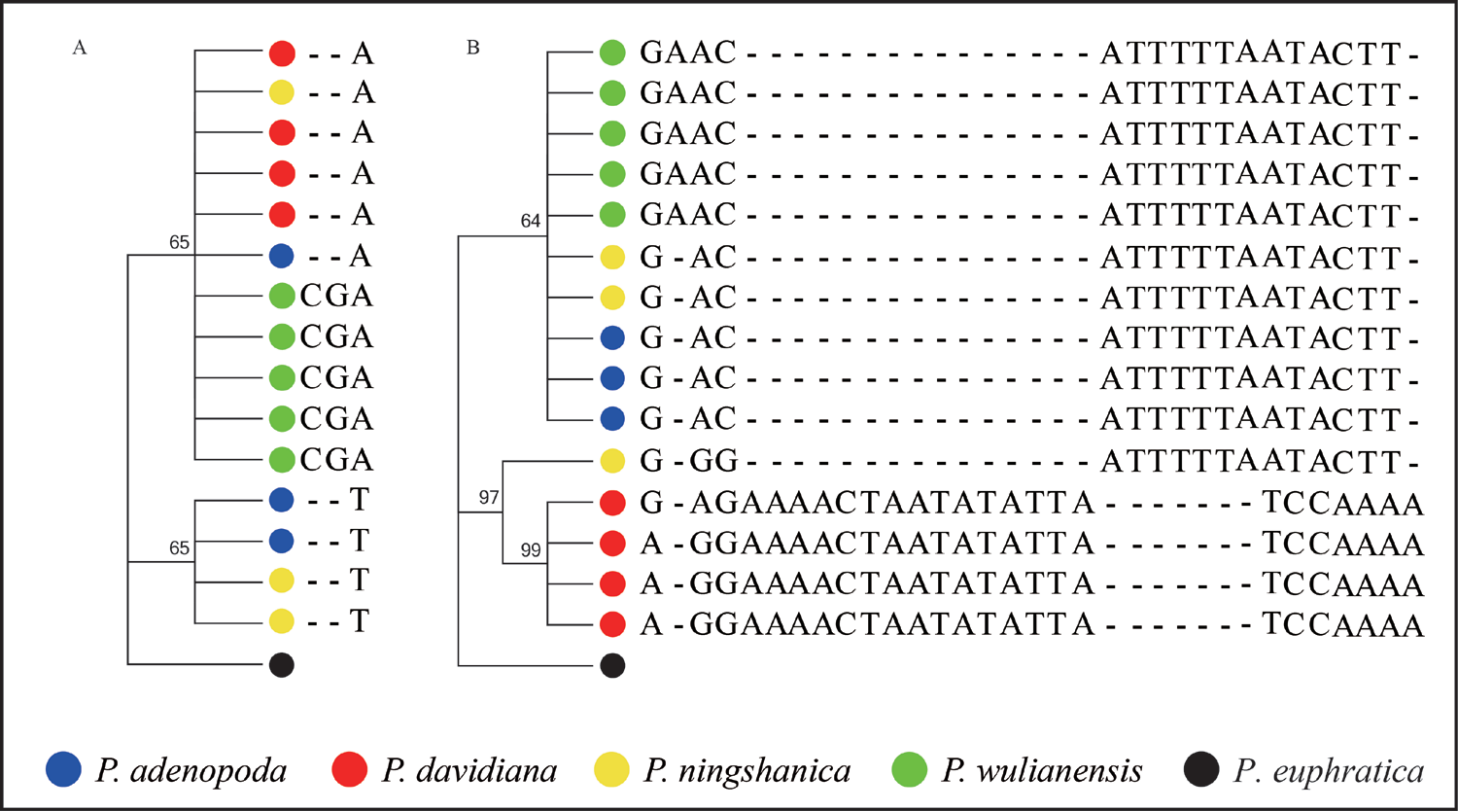
The neighbour-joining (NJ) tree of ITS variable sites (**A**); The neighbour-joining (NJ) tree of four cpDNA variable sites (**B**).

We have combined sequences of four cpDNAs for each individual into one cpDNA sequence. We aligned the cpDNA sequences of all individuals and identified 2, 1, 1 and 2 sequences for *P.davidiana*, *P.adenopoda*, *P.wulianensis* and *P.ningshanica*, respectively. The total length of the aligned cpDNA sequence was 1866 bp with 9 variable sites amongst different sequences from four species (Fig. 3B). NJ clustering of all different cpDNA sequences from four species similarly identified two tentative groups: one comprised of *P.davidiana* and *P.ningshanica*, while the other included those from *P.adenopoda*, *P.wulianensis* and *P.ningshanica*. We identified 1, 2, 1 and 2 different ITS sequences for the sampled individuals for *P.davidiana*, *P.adenopoda*, *P.wulianensis* and *P.ningshanica*. We aligned these ITS sequences from four species, which were 552 bps long with 1 variable site amongst all the different sequences from four species (Suppl. material S1: Table S3; Fig. 3A). NJ analyses of the ITS dataset identified two tentative groups: one comprised 4 sequences from *P.adenopoda* and *P.ningshanica* while the other, all four species.

## Discussion

Statistical analyses based on geometric morphometric measurements are highly successful at separating similar species (Villemant et al. 2007; Francuski et al. 2009), even when the individual character shows the overlapped variations between them (Lumley and Sperling 2010; Buck et al. 2012). Especially, geometric morphometrics could differentiate the overall changes in the gross morphology (Rohlf and Marcus 1993). Poplar leaves are ideal for geometric morphometric analyses, as they are two-dimensional, easily imaged and the venation provides many points that are clearly homologous and straightforward to landmark accurately. In addition, flower traits are highly static across the genus without variations and leaf characters are therefore used to classify different species (Dickmann and Stuart 1983; Eckenwalder 1996; Fang et al. 1999; Dickmann and Kuzovkina 2008). We tried to classify four popular species based on geometric morphometric analyses of leaf traits. Our results obviously suggested that *P.davidiana* and *P.adenopoda* differed distinctly from each other. *P.wulianensis* and *P.ningshanica* could not be distinguished from each other and they together clustered into one subgroup, which obviously belonged to the *P.adenopoda* group (Fig. 1B). Therefore, this statistical clustering indicated that both *P.wulianensis* and *P.ningshanica* may belong to the polymorphic *P.adenopoda*.

Genetic evidence, based on nuclear SSR loci, similarly recognised the distinct species boundary between *P.davidiana* and *P.adenopoda* (Fig. 2A, B). However, all sampled individuals of *P.wulianensis* belong to the *P.adenopoda* group without distinct introgression from *P.davidiana*. All sampled individuals of *P.ningshanica* shared similar genetic compositions, together belonging to the *P.adenopoda* group but with obvious genetic introgressions from the *P.davidiana* group. These individuals comprise the obvious backcrosses from *P.adenopoda*. Similarly, sequence variations from five DNAs (ITS, *matK*, *trnH-psbA*, *trnG-psbK* and *psbK-psbI*) seem to support these inferences. The connected sequences of four cpDNAs distinguished *P.davidiana* and *P.adenopoda* while all *P.wulianensis* individuals shared the same cpDNA sequences with *P.adenopoda*. We found two types of cpDNA sequences in *P.ningshanica* (Fig. 3B), clustering respectively with those from *P.davidiana* and *P.adenopoda*, which further suggested the hybrid origin of *P.ningshanica*. However, the initial hybrids must have repeatedly backcrossed with *P.adenopoda*, which resulted in the high genetic similarity of the sampled individuals of *P.ningshanica* to *P.adenopoda* but with introgression with *P.davidiana* (Fig. 3B). The interspecific hybrids in the genus *Populus* could be F1, F2 to multiple generation backcrossing hybrids (Braatne et al. 1992; Bradshaw et al. 2000; Feng et al. 2013; Jiang et al. 2016). We failed to find stable ITS differences between *P.davidiana* and *P.adenopoda*. It is highly probably that the gene flow, mediated by interspecific hybrids, had caused the concerted evolutions and indistinct differences in the ITS sequence variations (Feng et al. 2013; Jiang et al. 2016).

Overall, multiple lines (Figs 1B, 2B, C;) of evidence suggested that *P.wulianensis* was described based on the intraspecific variations of the polymorphic *P.adenopoda* and individuals ascribed to *P.ningshanica* are, in fact, hybrids between *P.adenopoda* and *P.davidiana* with the repeated backcrosses to the former. Both taxa should be treated accordingly in the taxonomic revision of the genus *Populus*.

**Additional specimens examined. China.** Anhui: Jiuhuashan mountain, on slope, 500 m elev., 18 Aug 1934 *C. S. Fan & Y. Y. Li 262* (NAS!). Huoshan county, on slope, 17 Apr 1959, *M. B. Deng & J. Q. Pan 0208* (NAS!). She county, in woods, 300 m elev., 04 May 1959, *S. She 1218* (NAS!). Jinzhai county, on slope, 12 Jul 1959, *Z. Jin 6044* (PE!). Jin county, in woods, 300 m elev., 10 Oct 1959, *Anonymous 793* (NAS!). Xiuning county, in roadside, 450 m elev., 29 Jun 1959, *R. H. Shan et al. 2661* (NAS!). Xuancheng city, in woods, 130 m elev., 02 Nov 1959 *Anonymous 262* (NAS!). ChongQing: Fengjie county, 860 m elev., 29 Apr 1959, *J. C. Zhang, 174* (SM!) Nanchuan county, 970 m elev., 13 Apr 1957, *G. F. Li 60474* (PE!). Nanchuan county, jin fo mountain, in forest edge, 1070 m elev., 20 Apr 1957, *J. H. Xiong & Z. L. Zhou 90383* (PE!). Qianjiang county, on slope, 980 m elev., 14 Aug 1988, *Z. C. Zhao, 88-1502* (PE!). Pengshui county, on slope, 800 m elev., 26 May 1959, *J. Z. Chuan, 03125* (PE!). Wushan county, 1080 m elev., 31 Mar 1958, *G. H. Yang, 57592* (PE!). Wushan county, huangniba mountain, 1100 m elev., 14 Apr 1958, *G. H. Yang 57715* (PE!). Wushan county, on slope, 1500 m elev., 17 May 1939, *T.P.Wang 10653* (PE!). Gansu: Wen county, 16 Oct 1958, *Z. P. Wei, 3047* (HIMC!). Wen county, 04 Apr 1964, *Z. B. Wang, 18862* (HNWP!). Guangxi: Longlin county, in woods, 1600 m elev., 09 Apr 1991, *H. Q. Wen 00375* (IBK!). Rongshui county, on slope, 1280 m elev., 20 Aug 1958, *S. Q. Chen 16359* (PE!). Tianyang county, 29 Nov 1978, *Z. Y. Chen 54101* (IBK!). Yangshuo county, 19 Apr 1956, *H. F Qin 700139* (IBK!). Guizhou: Dushan county, in grassland, 900 m elev., 24 Jul 1959, *Team of Libo 1198* (PE!). Guiding county, 400 m elev., 29 Jun 1930, *Y. Tsiang 5435* (IBSC!). Guiding county, in woods, 16 Jun 2014, *K. S. Mao & L. Zhang 2014-313* (SZ!). Guiyang city, Baiyun county, on slope, 1320 m elev., 22 Mar 2003, *M. T. An 5014* (PE!). Huangping county, in bushwoods, 1505 m elev., 04 May 1987, *J. M. Li 14* (GZTM!). Huishui county, on slope, 20 Jun 2014 *K. S. Mao & L. Zhang 2014-306* (SZ!). Luodian county, 300 m elev., 20 Mar 1960, *Z. S. Zhang & Y. T. Zhang 634* (IBSC!). Luodian county, in woods, 400 m elev., 22 Mar 1960, *Z. S. Zhang & Y. T. Zhang 133* (PE!). Pingtang county, in woods, 15 Jun 2014, *K. S. Mao & L. Zhang 2014-310* (SZ!). Qinglong county, in woods, 1600 m elev., 25 May 1987, *F. J. Li 403* (GZTM!). Suiyang county, in woods, 17 Jun 2014, *K. S. Mao & L. Zhang 2014-315* (SZ!). Wangmo county, in woods, 850 m elev., 01 Apr 2005, *G. F. Wang 1-1048* (PE!). Tongzi county, 23 May 1987, *K. M. Lan 870314* (GFS!). Yuqing county, in woods, 16 Jun 2014, *K. S. Mao & L. Zhang 2014-315* (SZ!). Zunyi county, in woods, 17 Jun 2014, *K. S. Mao & L. Zhang 2014-320* (SZ!). Henan: Luanchuan county, in woods, 28 Jun 2013, *K. S. Mao & L. Zhang 2013-078A* (SZ!). Nanyang city, funiu mountain, in woods, 1000 m elev., Jun 1959, *Anonymous 063* (HENU!). Tongbai county, tongbai mountain, on slope, 1000 m elev., 01 Apr 1960, *S. S. Kuang 468* (HENU!). Tongbai county, in woods, 27 Jun 2013, *K. S. Mao & L. Zhang 2013-063* (SZ!). Xixia county, 04 Agu 1956, *forestry department of Henan 27* (PE!). Hubei: Enshi city, in woods, 18 Jun 2013, *K. S. Mao & L. Zhang 2013-050* (SZ!). Hefeng county, 1250 m elev., 27 Aug 1958, *H. J. Li 5862* (PE!). Jianshi county, in woods, 23 Jun 2013, *K. S. Mao & L. Zhang 2013-048* (SZ!). Luotian county, in woods, 700 m elev., 10 Jul 1979, *Q. G. He 75-3* (PE!). Shennongjia, in woods, 06 Apr 1977, *Team of shennongjia 20635* (PE!). Xianfeng county, in woods, 25 Sep 1958, *H. J. Li 9252* (PE!). Xianfeng county, in woods, 22 Jun 2013, *K. S. Mao & L. Zhang 2013-053* (SZ!). Xinshan county, in woodlands, 1993 m elev., 27 March 2012, *D. G. Zhang 4383* (JIU!). Xinshan county, on slope, 1300 m elev., 14 May 1975, *Z. F. Fang et al 2005* (NAS!). Xinshan county, in woods, 20 Jun 2013, *K. S. Mao & L. Zhang 2013-046* (SZ!). Xuanen county, in woods, 22 Jun 2013, *K. S. Mao & L. Zhang 2013-052* (SZ!). Yun county, in woods, 20 Jun 2013, *K. S. Mao & L. Zhang 2013-038A* (SZ!). Hunan: Cili county, on slope, 840 m elev., 07 May 1986, *C. L. Peng 86040* (CSFI!). Dao county, on slope, 550 m elev., 04 May 1978, *Q. Z. Lin 0262* (CSFI!). Longshan county, 31 May 1958, *L. H. Liu 1885* (IBK!). Longshan county, in woods, 25 Jun 2013, *K. S. Mao & L. Zhang 2013-055* (SZ!). Luxi county, on slope, 400 m elev., 09 Apr 1982, *K. W. Liu 30045* (CSFI!). Sangzhi county, in woods, 25 Jun 2013, *K. S. Mao & L. Zhang 2013-061* (SZ!). Shimen county, 09 Jul 1979, *P. C. Cai 20198* (CSFI!). Shimen county, in woods, 420 m elev., 01 May 1980, *D. C. Xiao 80311* (CSFI!). Zhangjiajie city, zhangjiajie mountain, in woods, 870 m elev., 15 Apr 2015, *H. Zhou & D. S. Zhou 15041503* (CSFI!). Yizhang county, in woods, 09 Aug 1942, *S. Q. Chen 2107* (PE!). Yuanling county, in woods, 600 m elev., 22 Apr 1976, *Z. H. Shen 058* (CSFI!). Yuanling county, 600 m elev., 22 Apr 1976, *Anonymous 58* (IBSC!). Jiangxi: Lushan mountain, 15 May 1977, *C. F. Liang 34455* (IBK!). Tonggu county, 400 m elev., 06 Jun 1959, *J. Xiong 04268* (LBG!). Yushan county, 500 m elev., 14 Sep 1977, *S. K. Lai & H. R. Shan & D. F. Huang 039* (LBG!). Zhejiang: Chunan county, in broad-leaved forest, 700 m elev., 31 May 1959, *M. L. She 26991* (NAS!). Linan city, tianmu mountains in woods, 1 Oct 1934, J. *Shen 264* (NAS!). Linan city, tianmu mountain, on roadside, 430 m elev., 20 Jun 1983, *Q. X. Zheng S815-16* (PE!). Linan city, tianmu mountains in woods, 400 m elev., 22 Aug 1959, *Anonymous 28877* (NAS!). Taishun county, 25 May 2007, *Anonymous 24100* (HHBG!). Shaanxi: Foping county, in woods, 15 Jun 2013 *K. S. Mao & L. Zhang 2013-027A* (SZ!). Lueyang county, in valley, 600 m elev., 11 Nov 1989 *T. Y. Ding 2159* (IFP!). Mian county, 23 May 1942, *K.T.Fu 3508* (PE!). Nanzhen county, in woods, 15 Jun 2015 *L. Zhang 2015-19* (SZ!). Pingli county, on slope, 550 m elev., Apr 1959,*Y. L. Qiao, 1114* (PE!). Shiquan county, in woods, 15 Jun 2013, *K. S. Mao & L. Zhang 2013-033B* (SZ!). Xixiang county, on slope, 650 m elev., 08 Apr 1958, *J. Q. Xing 18* (NAS!). Xixiang county, in woods, 16 Jun 2013, *K. S. Mao & L. Zhang 2013-032* (SZ!). Shandong: Kunyushan mountain, 12 Jul 1957, *Anonymous 3095* (IBSC!, PE!). Kunyushan mountain, in woods, 188 m elev., 12 May 2016, *L. Zhang 2016300* (SZ!). Sichuan: Cangxi county, 1070m elev., 08 May 1959, *Z. S. Qin 02663* (CDBI!). Da county, 800 m elev., 23 Feb 1979, *Team of Bazhong 830* (SM!). Da county, 1000 m elev., 20 Aug 1978, *Team of Kaijiang 706* (SM!). Dujiangyan city, in valley, 1200 m elev., 11 May 1930, *F. T. Wang 20749* (PE!). Emeishan mountain, on slope, 400 m elev., 03 Apr 1940, *W. P. Fang 13968* (WUK!). Jiangjin county, 1100 m elev., 26 Jul 1978, *Team of Dazu 584* (SM!). Jiulong county, on slope, 1000 m elev., 03 May 1959, *M. X. Wang 7680* (PE!). Leibo county, 1100 m elev., Jun 1963, *Z. T. Guan 373* (IBSC!). Mabian county, 1000 m elev., 31 May 1962, *Q. L. Zhang 10123* (IBSC!). Qingchuan county, in woods, 10 May 2015, *L. Zhang 201501* (SZ!). Tianquan county, on slope, 950 m elev., 14 Sep 1963, *K. J. Guan & W. C. Wang 3470* (PE!). Tongjiang county, on roadside, 1900 m elev., 19 Sep 1978, *Team of Tongjiang 1385* (SM!). Guangyuan city, on roadside, 1720 m elev., 08 Jul 1978, *Team of Guangyuan 0880* (SM!).

***Populus×ningshanica*** C. Wang & Tung in Journal of Beijing Forestry University 4: 19. 1979. TYPE: China (holotype, WUK not seen).

**Additional specimens examined. China.** Gansu: Wen county, 660 m elev., 15 Jun 2015 *L. Zhang & Z. Q. Wang, 01014868* (SZ!). Shaanxi: Lueyang county, 770 m elev., 10 Jun 2015, *L. Zhang & Z. Q. Wang, 01014866* (SZ!).

## Supplementary materials

Supplementary material 1 Tables S1–S3 Data type: molecular data Explanation note: **Table S1.** Details for the 14 microsatellite loci adopted in genetic survey. **Table S2.** Details for the four chloroplast DNA fragments adopted in genetic survey. **Table S3.** Length and variations for each DNA region and for the combination of the four plastid regions This is an open access article distributed under the terms of the Creative Commons Attribution License (CC BY 4.0), which permits unrestricted use, distribution, and reproduction in any medium, provided the original author and source are credited.Lei Zhang, Mingcheng Wang, Tao Ma, Jianquan Liu
