# Supplementary material for: Taxonomic status of Populuswulianensis and P.ningshanica (Salicaceae)
Source: PhytoKeys. 2018 Sep 10;(108):117–29. doi: 10.3897/phytokeys.108.25600 (PMC6160800; doi:10.3897/phytokeys.108.25600)
Supplement: Supplementary material 1 — Tables S1–S3 [file phytokeys-108-117-s001.doc]

Table S1. Details for the 14 microsatellite loci adopted in genetic survey

| Locus | Primer sequence (5'-3') | Repeats | Length（bp） | Annealing temp(℃) |
| --- | --- | --- | --- | --- |
| GCPM_124 | TTTGAGCACTTCAACTACCA | (CAC)_6_ | 198 | 55 |
|  | TGTCTTCCCTTAGTCACCAC |  |  |  |
| GCPM_1063 | AGTTAATTGCGCATGTTCTT | (CA)_16_ | 165 | 55 |
|  | AAACAAACTCCAGCAAACAT |  |  |  |
| GCPM_1158 | ATGCACTTCCTTCCAAATTA | (CTG)_6_ | 225 | 55 |
|  | ATCAGTTCCTTCAGCTTCAA |  |  |  |
| ORPM_190 | CCCTGGTTTTCTCTTCTTGG | (TG)_7_ | 209 | 55 |
|  | CCAGATTGGACTTGGGATTC |  |  |  |
| PeuSSR_56336 | TCAGAAGACCCAACCAGAT | (AT)_13_ | 348-334 | 55 |
|  | GTCATGAGATGCGTTTGC |  |  |  |
| PeuSSR_83115 | AGCTCCATGGAAAAGCAAC | (AG)_11_ | 311-337 | 55 |
|  | AGATGTGAAGAGATGGTGTTTAC |  |  |  |
| PeuSSR_104279 | TGAGAAAGAAGCAACAATGTG | (GA)_13_ | 341-329 | 56 |
|  | GTCACCTGTCTCCTAATGAAAAAC |  |  |  |
| PeuSSR_104938 | GAAAAAGGCGAACCATTCAAAG | (GA)_19_ | 463-451 | 60 |
|  | TGGATATTTTGGTGCTTGTGAGT |  |  |  |
| PeuSSR_135862 | TGTCTTGGCTTAAACCTCC | (AGA)_11_ | 272 | 55 |
|  | CCACTCCATTTTCCCTATCCTCTA |  |  |  |
| PeuSSR_149476 | CTGTTACCTGGCATTCTGTATCA | (TC)_7_ | 313 | 59 |
|  | TACACTGGGAGCATTAGGCAG |  |  |  |
| PeuSSR_174794 | TCTCTGTCCTTTTTGAGGTCTG | (TAA)_10_ | 230-215 | 58 |
|  | GGCAGAATACGCAAGGGATGA |  |  |  |
| PeuSSR_209119 | CGTGACGAAAGCTGGTATTTTCTT | (TC)_19_ | 463-447 | 60 |
|  | GGATTCGTACCAGTGAGTGTGGT |  |  |  |
| GCPM_1260 | CACAGGAACCTGGTTATCAT | (TG)_11_ | 134 | 55 |
|  | CTGGCATTCCTTCTAAGCTA |  |  |  |
| PeuSSR_48175 | AGAATATCGCCATTTATGACCT | (TG)_6_ | 222-242 | 56 |
|  | CTTCCACAACCCCTCTCA |  |  |  |

**Table** S2**.** Details for the four chloroplast DNA fragments adopted in genetic survey

| **Region** | **Successful primers and their sequences (5’-3’)** | | **References** |
| --- | --- | --- | --- |
| ITS | F | CGTAGCTACTTCTTCGCAGC | White et al., 1990 |
|  | R | CCTTATCATTTAGAGGAAGGAG | White et al., 1990 |
| *mat*K | F | TAATGAGAAAGATTTCTGCATATACG | Schroeder *et al.*, 2012 |
|  | R | TTTACGATCAATTCATTCAATATTTCC | Schroeder *et al.*, 2012 |
| *trnH-psbA* | F  R | CGCGCATGGTGGATTCACAATCC  GTTATGCATGAACGTAATGCTC | Sang et al., 1997  Tate & Simpson, 2003 |
| *trn*G-*psb*K | F | GAAGGATTCGAACCTCCGAATG | Schroeder *et al.*, 2012 |
|  | R | CTGGCATAACATCTACGATTGG | Schroeder *et al.*, 2012 |
| *psb*K-*psb*I | F | CCAATCGTAGATGTTATGCCAG | Schroeder *et al.*, 2012 |
|  | R | GGATTACGCCCTGGATCATTAG | Schroeder *et al.*, 2012 |

Reference:

White TJ, Bruns T, Lee S, Taylor J (1990) Amplification and direct sequencing of fungal ribosomal RNA genes for phylogenetics. PCR protocols: a guide to methods and applications.

Schroeder H, Hoeltken A, Fladung M (2012) Differentiation of Populus species using chloroplast single nucleotide polymorphism (SNP) markers-essential for comprehensible and reliable poplar breeding. Plant Biology 14(2): 374-381.

Sang TQ, Crawford D, Stuessy.T (1997) Chloroplast DNA phylogeny, reticulate evolution, and biogeography of *Paenoia* (paenoiaceae). American Journal of Botany 84(8):1120-1136.

Tate JA, Simpson BB (2003) Paraphyly of *Tarasa* (Malvaceae) anddiverse origins of the polyploid species. Systematic Botany 28(4): 723-737.

Table S3. Length and variations for each DNA region and for the combination of the four plastid regions

| Region | Seq.length（bp） | No.SNPs | %SNP | NO.InDels |
| --- | --- | --- | --- | --- |
| ITS | （550-）552 | 1 | 0.18 | 2 |
| *matK* | 772 | 1 | 0.13 | 0 |
| *psbK-psbI* | （477-）478 | 2 | 0.42 | 1 |
| *trnG-psbK* | （461-）469 | 0 | 0.00 | 22 |
| *trnH-psbA* | （139-）140 | 6 | 4.29 | 1 |
| Four cpDNA regions | （1849-）1859 | 9 | 0.48 | 24 |
